# Organizational theory for dissemination and implementation research

**DOI:** 10.1186/s13012-017-0592-x

**Published:** 2017-05-12

**Authors:** Sarah A. Birken, Alicia C. Bunger, Byron J. Powell, Kea Turner, Alecia S. Clary, Stacey L. Klaman, Yan Yu, Daniel J. Whitaker, Shannon R. Self, Whitney L. Rostad, Jenelle R. Shanley Chatham, M. Alexis Kirk, Christopher M. Shea, Emily Haines, Bryan J. Weiner

**Affiliations:** 1 Department of Health Policy and Management, Gillings School of Global Public Health, The University of North Carolina at Chapel Hill, 1103E McGavran-Greenberg, 135 Dauer Drive, Campus Box 7411, Chapel Hill, NC 27599-7411 USA; 2 College of Social Work, The Ohio State University, 1947 College Road, Columbus, OH 43210 USA; 3 Department of Health Policy and Management, Gillings School of Global Public Health, The University of North Carolina at Chapel Hill, 1105C McGavran-Greenberg, 135 Dauer Drive, Campus Box 7411, Chapel Hill, NC 27599-7411 USA; 4 Department of Health Policy and Management, Gillings School of Global Public Health, The University of North Carolina at Chapel Hill, 1107C McGavran-Greenberg, 135 Dauer Drive, Campus Box 7411, Chapel Hill, NC 27599-7411 USA; 5 Department of Maternal and Child Health, The University of North Carolina at Chapel Hill, 401 Rosenau Hall, Campus Box 7445, Chapel Hill, NC 27599-7445 USA; 6 Department of Family Medicine, University of Calgary, 8th Floor, Sheldon M. Chumir Health Centre, 1213 - 4 Street SW, Calgary, Alberta T2R 0X7 Canada; 7 School of Public Health, Georgia State University, PO Box 3995, Atlanta, GA 30302-3995 USA; 8 National SafeCare Training and Research Center, Mark Chaffin Center for Healthy Development, PO Box 3995, Atlanta, GA 30302-3995 USA; 9 Department of Health Policy and Management, Gillings School of Global Public Health, The University of North Carolina at Chapel Hill, 1104F McGavran-Greenberg, 135 Dauer Drive, Campus Box 7411, Chapel Hill, NC 27599-7411 USA; 10 Department of Global Health, University of Washington, Box 357965, Seattle, WA 98195-7965 USA; 11 Department of Health Services, University of Washington, Box 357965, Seattle, WA 98195-7965 USA

**Keywords:** Organizational theory, External environment, Adoption, Implementation, Sustainment

## Abstract

**Background:**

Even under optimal internal organizational conditions, implementation can be undermined by changes in organizations’ external environments, such as fluctuations in funding, adjustments in contracting practices, new technology, new legislation, changes in clinical practice guidelines and recommendations, or other environmental shifts. Internal organizational conditions are increasingly reflected in implementation frameworks, but nuanced explanations of how organizations’ external environments influence implementation success are lacking in implementation research. Organizational theories offer implementation researchers a host of existing, highly relevant, and heretofore largely untapped explanations of the complex interaction between organizations and their environment. In this paper, we demonstrate the utility of organizational theories for implementation research.

**Discussion:**

We applied four well-known organizational theories (institutional theory, transaction cost economics, contingency theories, and resource dependency theory) to published descriptions of efforts to implement SafeCare, an evidence-based practice for preventing child abuse and neglect. *Transaction cost economics* theory explained how frequent, uncertain processes for contracting for SafeCare may have generated inefficiencies and thus compromised implementation among private child welfare organizations. *Institutional theory* explained how child welfare systems may have been motivated to implement SafeCare because doing so aligned with expectations of key stakeholders within child welfare systems’ professional communities. *Contingency theories* explained how efforts such as interagency collaborative teams promoted SafeCare implementation by facilitating adaptation to child welfare agencies’ internal and external contexts. *Resource dependency theory* (RDT) explained how interagency relationships, supported by contracts, memoranda of understanding, and negotiations, facilitated SafeCare implementation by balancing autonomy and dependence on funding agencies and SafeCare developers.

**Summary:**

In addition to the retrospective application of organizational theories demonstrated above, we advocate for the proactive use of organizational theories to design implementation research. For example, implementation strategies should be selected to minimize transaction costs, promote and maintain congruence between organizations’ dynamic internal and external contexts over time, and simultaneously attend to organizations’ financial needs while preserving their autonomy. We describe implications of applying organizational theory in implementation research for implementation strategies, the evaluation of implementation efforts, measurement, research design, theory, and practice. We also offer guidance to implementation researchers for applying organizational theory.

## Background

Over the past decade, the field of implementation science has grown rapidly, with a devoted international journal, new funding mechanisms, and other implementation-related initiatives around the world. These investments highlight the complexities of making change in real-world care settings. We have learned, for example, how strong leadership [1, 2], supportive organizational culture and climate [3], clinician buy-in and receptivity to change [4], and robust consultation and supervision [5, 6] are all critical for implementation success.

Yet, even with optimal internal organizational conditions, implementation can be undermined by changes in organizations’ external environments, such as fluctuations in funding, adjustments in contracting practices, new technology, new legislation (e.g., requirements set forth in the Affordable Care Act), changes in clinical practice guidelines and recommendations, or other environmental shifts [7–11]. *Internal* organizational conditions are increasingly reflected in implementation frameworks, focusing on intra-organizational or inner context constructs like structure, leadership, and social context. However, nuanced explanations of how organizations’ *external* environments influence implementation success are generally lacking in implementation research [12–15].

Organizational theories offer implementation researchers a host of existing, highly relevant, and heretofore largely untapped explanations of the complex interaction between organizations and their environment. Organizational theories describe, explain, and predict the complex interaction between organizations and their external environments. Thus, these theories have potential to explain and ground investigations focused on the role of policies, institutions, funding fluctuations, contract design, procurement processes, and workforce dynamics in implementation.

With roots in management and sociology [16], organizational theories have been used to explain phenomena in the fields of education, human services, public management, and health services research [17–20]. Organizational sociologists have called for the use of organizational theory in research on healthcare organizations [21] and for a greater focus on organizations in implementation science [22]. Yano [22] suggested that implementation researchers could leverage organizational theory to identify organization-level implementation determinants; however, Yano’s primary objective was to advocate for the use of organization-level implementation determinants—not organizational theory—in implementation research. With a few notable exceptions [23–27], the application of organizational theory in implementation research remains limited. To date, organizational theory remains unfamiliar to many implementation researchers, and guidance for applying organizational theory in implementation research is lacking. To advance rigorous and theoretically grounded studies of the influence of outer context on implementation, in this paper, we discuss how four organizational theories apply to implementation research.

In the “Discussion” section, we illustrate the use of four organizational theories (transaction cost economics, resource dependency theory, institutional theory, and contingency theories) that we found to be particularly apt for retrospectively explaining how and why interactions between organizations and their external environments influenced the implementation of SafeCare, a program for preventing child maltreatment. In the “Summary” section, we describe how organizational theories can be used prospectively, to inform development and empirical tests of, for example, policy and financing strategies.

## Discussion

### SafeCare®

SafeCare® is an evidence-based behavioral parent training model that targets the proximal parenting behaviors that lead to the abuse and neglect of children with at-risk (typically involved in child welfare or intensive prevention settings) parents of children 0 to 5 years old [28]. Using a structured, behavioral approach, trained SafeCare providers deliver in-home parent training in three core content areas: home safety, child health, and parent-child interactions. The program originated in 1979 in a child welfare system in rural southern Illinois as Project 12-Ways, which trained parents in up to 12 skill-sets [29]. Because of its many components, Project 12-Ways was not easily disseminated, and thus, it was not feasible to take the program to scale. Accordingly, the program was condensed to three skill-sets and renamed SafeCare. SafeCare is typically delivered in 18 weekly sessions (6 sessions per module), with sessions averaging 1 hour.

SafeCare in its current form has been implemented in 23 US states—6 of which executed statewide or large regional rollouts—and 6 other countries (Belarus, Spain, Israel, England, Australia, and Canada). SafeCare has been implemented in a variety of service settings, including child welfare, prevention, judicial, and early childhood education. Within US child welfare settings, interventions may be implemented by public child welfare agencies (state- or county-level authorities responsible for child safety, permanency, and well-being), by private child welfare organizations (local private community-based organizations (CBOs) contracted by public child welfare agencies to deliver an array of services to children and families), or throughout the entire state or county child welfare system (which includes the public agency and its network of private organizations) [30, 31]. The complex organizational relationships inherent to this public-private child welfare system provide an ideal context for applying organizational theories to understand the influence of the external environment in the implementation of SafeCare. The model has been evaluated with various research designs, including single-case studies, quasi-experimental studies, and randomized trials [32]. The largest randomized trial was a statewide trial in the Oklahoma child welfare system that compared SafeCare to home-based services that did not include SafeCare [33]. The findings indicated that SafeCare significantly reduced child welfare recidivism [33], findings which were consistent across racial and ethnic groups, including a subsample (*N* = 354) of American Indians [34]. Finally, other research has demonstrated that SafeCare positively impacts parent engagement and retention in voluntary prevention services [35], parent satisfaction, perceived cultural competency of the program [33], and caseworker retention [36] compared to service as usual in child welfare and prevention systems.

The National SafeCare Training and Research Center (NSTRC) provides a variety of supports to implementing agencies to train providers to deliver SafeCare with fidelity. Providers are typically either bachelor’s- or master’s-degreed professionals and are employed by an agency that contracts with state or county government to deliver child welfare or prevention services. In some cases, the providers of SafeCare are county or state employees, as states have different models for providing services to families, although the trend is largely toward private CBOs. All agencies that contract with the NSTRC for SafeCare training and implementation support begin the partnership with implementation planning discussions about resources, eligible client populations, and general readiness. Once a contract is established for training, NSTRC faculty and staff conduct a general readiness process, which includes an on-site orientation for trainees, leadership, and relevant community partners (e.g., referring agencies, funders) to finalize the implementation and training plan. Subsequently, SafeCare providers attend a 4-day training on delivering the SafeCare model to families. Once they begin to implement SafeCare, providers receive coaching by NSTRC trainers. Coaching consists of fidelity observations (primarily through audio recordings), followed by coaching sessions to discuss positive and corrective feedback from a SafeCare coach. The frequency of coaching is front-loaded with sessions occurring weekly until the provider is certified (i.e., has demonstrated fidelity in three sessions in each of the three modules) and then is conducted monthly [37]. Implementation support also includes ongoing team and administrative meetings to ensure fidelity, address implementation challenges, and optimize sustainment.

Despite demonstrated effectiveness and available implementation supports, challenges have been documented related to SafeCare *adoption* [10] (the decision to use SafeCare), *implementation* [38] (the complex process whereby SafeCare use becomes increasingly proficient and consistent among organizational members), and *sustainment* [39–41] (a state in which SafeCare use persists over several years without SafeCare developers’ assistance). In the sections that follow, we describe challenges associated with these three stages of SafeCare life cycle (adoption, implementation, and sustainment). The SafeCare studies that we cite in this paper were conducted by established implementation and intervention scholars, including those who have expertise in organizational theory and behavior. However, their studies were designed to focus on features and processes within internal organizational environments (e.g., community development teams, leadership). Thus, organizational theories fell outside the scope of the studies. In collaboration with SafeCare experts (DJW, SRS, WLR, JRSC), we propose in this paper that organizational theories can be leveraged to *retrospectively* explain how relationships between organizations and their external environments contributed to their success adopting, implementing, and sustaining SafeCare. Table 1 summarizes the main propositions of the four organizational theories that we feature in the paper and their application to SafeCare. (Note that the organizational theories featured in this paper are significantly more complex than we are able to include in the table or the descriptions that follow.) In the “Summary” section, we argue that these organizational theories offer unique insights into adoption, implementation, and sustainment that could advance implementation research *prospectively*.

**Table 1:** Organizational theory descriptions and applications to SafeCare

Theory	Main propositions	Applications to SafeCare
Transaction cost economics	• Transaction costs influence whether an organization decides to contract with another organization to implement an EBI. • Decreases in transaction frequency will increase the likelihood that organizations will contract with other organizations to implement an EBI. • Past relationships between organizations reduce the uncertainty and costs associated with contracting.	Adoption: • Child welfare systems’ decision to contract with CBOs to administer EBIs rather than acting as direct EBI providers internally was likely influenced by costs. • The cost of EBI administration is driven by the frequency of collaboration between CBOs and child welfare systems and the familiarity of child welfare systems with CBOs.
Institutional theory	• Organizations implement EBIs that are viewed as legitimate by institutions within their environment. • Organizations adopt certain EBIs in response to coercion or strong pressures to comply with rules, mandates, and regulations. • Organizations mimic the behaviors and structures of other successful organizations such as adoption of certain EBIs. • Organizations will adopt EBIs that align with professional norms.	Adoption: • Child welfare systems’ decision to adopt SafeCare was likely influenced by pressure from policymakers to provide EBIs, perceptions that SafeCare was viewed as a norm, and advocacy from child welfare professional communities for use of SafeCare. Sustainment: • Efforts to maintain SafeCare contracts may have coerced CBOs to sustain SafeCare by establishing rules, regulations, and mandates set forth in contracts. • The contracts garnered support for SafeCare, creating normative pressure on CBOs to sustain SafeCare.
Contingency theories	• Organizations’ design decisions are contingent upon the organization’s internal and external contexts. • Successful EBI implementation is influenced by whether the EBI fits with an organization’s internal context. • Organizations’ ability to adapt to their external context influences successful EBI implementation.	Implementation: • The use of ICTs allowed child welfare systems to respond to external contexts such as local client needs. • Internal context influenced implementation as larger, governmental organizations had less flexibility in how SafeCare could be implemented.
Resource dependency theory	• Organizations’ design decisions are informed by their dependence on other organizations, ability to maintain autonomy, and relationships with other organizations. • Organizations form relationships with other organizations to acquire and maintain resources and autonomy.	Implementation: • CBOs depended on the organizations that funded them and SafeCare developers (for expertise), which lessened their autonomy and power. • CBOs often negotiated the balance of autonomy and dependence on other organizations by establishing relationships via ICTs, which minimized the resources individual CBOs needed to implement SafeCare. Sustainment: • Policymakers could have earmarked funds for contracts that would have supported SafeCare to obtain sufficient resources for SafeCare sustainment. • Train-the-trainer models decreased CBOs’ dependence on SafeCare developers so that their staff could autonomously sustain the practice without.

*CBO* community-based organization, *EBI* evidence-based intervention, *ICT* interagency collaborative team

### Adoption

Adoption refers to an individual or organizational decision to begin using an innovation. In the USA, child welfare systems encounter pressure from policymakers and funders to address problems with child abuse and neglect. Increasingly, child welfare systems are encouraged to adopt and implement evidence-based interventions (EBIs), such as SafeCare, to improve child safety, permanency, and well-being. Recent trends are for state and county public welfare agencies to privatize services by contracting with private CBOs to deliver EBIs, including SafeCare, rather than adopting the models for use “in-house” [31]. Transaction cost economics theory and institutional theory, two widely used organizational theories, are particularly apt for explaining (1) why public child welfare agencies increasingly contract with CBOs to administer EBIs to prevent child abuse and neglect, and (2) why the EBI of choice was often SafeCare.

#### Why did public child welfare agencies contract with CBOs to administer EBIs to prevent child abuse and neglect? A transaction cost economics explanation

Ostensibly, public child welfare agencies had the option of acting as direct service providers, hiring staff to administer SafeCare. Instead, many public child welfare agencies contract with CBOs to administer SafeCare [31]. To improve efficiency, responsiveness, and flexibility in the services provided, many child welfare agencies in the USA have become increasingly privatized by increasing contracting with private CBOs to deliver core child welfare services, such as maltreatment prevention [31, 42, 43]. *Transaction cost economics* (TCE) theory explains how public child welfare agencies’ decisions to adopt SafeCare may be influenced by their ability to “buy” SafeCare by contracting with CBOs instead of “making” SafeCare (i.e., administering it in-house), diminishing the cost of administering SafeCare. TCE suggests that costs are driven by the transactions required to accomplish tasks. TCE states that three factors influence the cost of a transaction and, in turn, the “make” vs. “buy” decision: (1) frequency of the transaction, (2) uncertainty of the transaction, and (3) asset specificity required for the transaction [44]. Transaction frequency refers to how often parties engage in a transaction; increased transaction frequency increases transaction costs. For example, as described in detail below, changes in the procurement process used by public child welfare agencies increased the frequency with which they had to communicate with CBOs to contract for SafeCare, thus increasing the cost of contracting with CBOs for SafeCare. Uncertainty refers to information regarding the transaction that is unclear or unknown, thus increasing transaction costs. This would be the case if child welfare agencies had little information regarding SafeCare, increasing the cost of adoption. Asset specificity refers to how specialized resources must be for the transaction to transpire; increased asset specificity increases transaction costs. For example, staff who are specially trained in SafeCare represent a highly specialized and potentially non-transferrable asset, thus increasing the cost associated with adopting SafeCare. Public agencies might be more likely to contract with CBOs to deliver SafeCare when that partnership is expected to yield fewer costs than “making” the service in-house. For instance, in the case of SafeCare, adopting agencies would need to invest in specialized training, materials, and fidelity monitoring to administer SafeCare in-house. In addition, an agency might need to hire new staff or divert existing staff away from other duties for SafeCare training and service delivery. The cost of investing in these assets that are highly specific to SafeCare and might not transfer to other areas of agency functioning may make agencies more likely to contract with CBOs to deliver SafeCare than “making” the service in-house. Although contracting for services can also be costly (e.g., in terms of costs associated with the search for a CBO to contract with for SafeCare implementation and contract management), contracting could be less expensive and offer more flexibility to agencies than administering SafeCare in-house, facilitating SafeCare adoption. Thus, many child welfare agencies contract with CBOs to deliver services.

TCE may explain the stagnation in EBI adoption [45]: if the costs of administering SafeCare in-house and contracting with CBOs to do so are both too high, public child welfare agencies may have opted out of adopting SafeCare altogether. Indeed, as Willging et al. [10] documented, changes in the procurement process used by public child welfare agencies increased the transaction costs associated with contracting with CBOs for SafeCare, potentially deterring them from adopting SafeCare altogether. In the traditional contract bidding system, public child welfare agencies were not blinded to bidding CBOs. In fact, public child welfare agencies had established relationships with CBOs characterized by relatively infrequent negotiations and certainty around the feasibility of the CBO administering a high-quality EBI. Specifically, some CBOs with which public child welfare agencies had historically contracted engaged in interagency collaborations to support the administration of SafeCare, an EBI with which CBOs had substantial and increasing familiarity. These collaborations decreased CBOs’ costs of administering the EBI. Taken together, the traditional contract bidding system minimized the frequency of transactions between public child welfare agencies and CBOs, and it minimized the uncertainty of the transactions for EBI contracts. In contrast, a new procurement system, Best Value-Performance Information Procurement System (BV-PIPS), introduced a blind bidding process that produced substantial uncertainty around contracting. This process increased the frequency of transactions between child welfare systems and CBOs due to lack of familiarity, making the cost of contracting with CBOs to administer an EBI greater than the traditional contract bidding system.

#### Institutional theory explains why SafeCare was often the EBI selected to prevent child abuse and neglect among CBOs

*Institutional theory* suggests that organizations, including the public and private agencies that constitute child welfare systems, are motivated to align their structures and behaviors with the values, norms, and expectations espoused by institutions in their environments (e.g., government, client groups, accrediting bodies), as opposed to being primarily motivated by demonstrating superior outcomes or performance, to maintain legitimacy [46, 47]. Institutional theory posits that organizational changes, such as the adoption of SafeCare, are a consequence of three types of institutional pressures: coercive, mimetic, and normative isomorphism. Organizations change as an adaptive response to coercion, or strong pressures to comply with rules, regulations, and mandates. For example, organizations adopt new forms, technologies, or behaviors to adhere to mandates from government agencies [48, 49]. This was likely the case for child welfare systems. Policymakers, including state and county administrators in child welfare agencies, demand solutions to problems with child abuse and neglect; in Oklahoma, for example, policymakers specifically advocated for the use of SafeCare, making it the most politically viable mechanism for child welfare systems to prevent child abuse and neglect [50]. Further, federal and state legislators increasingly require public child welfare agencies or their contractors to use EBIs. In the USA, Washington state legislature passed several bills encouraging the use of EBIs [51, 52], and at the federal level, the 2016 Families First Prevention Services Act (HR5456; not enacted) [53] would have restricted funding to evidence-based child welfare interventions only. These efforts represent coercion to adopt SafeCare and the handful of other evidence-based child welfare interventions.

It is also likely that SafeCare was often the EBI of choice to prevent child abuse and neglect because implementing EBIs is increasingly viewed as a norm among child welfare systems [53], despite persistent confusion regarding EBIs and how to effectively implement them. Institutional theory posits that, to gain legitimacy and resources, organizations mimic the behaviors or structures of other successful organizations [54], especially under conditions of uncertainty or goal ambiguity. Policymakers in Oklahoma reported that they supported SafeCare implementation because they viewed its designation as an EBI—a designation that was upheld by peer organizations as legitimate—as assurance that it might produce favorable outcomes [50]. Further, among the contracted CBOs, some engaged in interagency collaborative teams (ICTs) to administer SafeCare [55]; among these ICTs, SafeCare may have been viewed as the normative EBI for preventing child abuse and neglect, representing additional normative pressure from CBOs.

Institutional theory also posits that organizations adapt and align with strong professional norms, values, and expectations conveyed via formal education, training, licensing, and accrediting bodies. Few child welfare professionals likely undergo formal training in child welfare interventions prior to entering the workforce because they tend to come from disciplines outside of social work [56]; however, normative pressures are likely salient for the child welfare systems targeted for SafeCare given that they rely on workers who are professionalized and therefore subject to influence from professional organizations and normative pressures of the field. Hurlburt et al. found that leadership within child welfare systems’ professional communities, including directors of community-based provider organizations and local foundations, advocated for the use of SafeCare [57].

#### Organizational theories’ contribution to understanding SafeCare’s adoption

The conclusions that authors have drawn regarding SafeCare’s adoption may be enhanced by drawing on TCE and institutional theory. For example, Willging et al. concluded that a substantial barrier to SafeCare adoption was the costs of training, materials, fidelity monitoring, and clinicians’ time away from their clinical duties [50]. Viewed through the lens of TCE, these discrete variables are examples of a host of potential determinants of adoption. Training, materials, etc. represent costs associated with a broader transaction required to administer SafeCare. Public child welfare agencies minimize and address such cost-related barriers to adoption by contracting with private CBOs to deliver EBIs like SafeCare. These barriers are minimized insofar as the transaction costs associated with contracting are also minimal. Especially within human services, these contracting relationships are based on familiarity and trust, which serves as a safeguard for both the public child welfare agency and the private CBOs, reducing the risk (and costs) associated with the contract, thus promoting adoption of the EBI. BV-PIPS, a blind process, exacerbated uncertainty and risks for both the public agency and private CBOs, heightening transaction costs, thus disrupting SafeCare’s adoption. In this sense, TCE explains how BV-PIPS disrupted SafeCare’s adoption by increasing the contracting costs.

Institutional theory offers another explanation for why BV-PIPS had the capacity to disrupt SafeCare’s adoption among the private CBOs. Traditionally, the default EBI for public child welfare agencies and CBOs was SafeCare, due to support in the form of coercive pressure from policymakers, mimetic pressure in the form of pervasive ICTs around SafeCare, and normative pressure in the form of general professional acceptance of SafeCare as a viable approach to preventing child abuse and neglect. In contrast, BV-PIPS allowed bidders greater choice of EBIs. In fact, Willging et al. found that many CBOs bidding under BV-PIPS did not propose to use SafeCare [10], and many proposed using interventions that were not evidence-based; this may speak to the limits of institutional pressure to adopt EBIs in the face of the more substantial transaction costs associated with contracting under BV-PIPS.

### Implementation

Implementation refers to the complex process whereby targeted organizational members’ use of an innovation such as SafeCare becomes increasingly proficient and consistent. Child welfare systems that adopted SafeCare faced conditions that may have limited the proficiency and consistency of SafeCare use. Specifically, the CBOs contracted to administer SafeCare exhibited unique local needs including varying cultures and client demographics. CBOs also had limited time, staff, funding, and other resources available to implement SafeCare. To address these potential barriers to implementation, some CBOs engaged in ICTs. ICTs were formed with an explicit goal of developing locally based SafeCare trainers. The SafeCare developers (NSTRC) trained an initial group of 12 staff from several different local organizations; those staff members were trained to a high degree of expertise, and eventually, the best performers were trained as SafeCare trainers. Subsequently, the local SafeCare trainers trained other members of other organizations and conducted coaching and implementation support [55]. The following sections will apply two additional organizational theories—contingency theories and resource dependency theory (RDT)—to explain how ICTs addressed local CBO needs and minimized CBOs’ resource constraints, thereby facilitating SafeCare implementation.

#### How did ICTs address local CBO needs, thereby facilitating SafeCare implementation? A contingency theories perspective

ICTs’ ability to address local CBO needs, thereby facilitating SafeCare implementation, may be explained using contingency theories. *Contingency theories* are a class of behavioral theories that state, in essence, that optimal structure and leadership is contingent upon an organization’s internal and external contexts: An organization that is effective and efficient under some conditions may not be successful in others [58, 59]. One of implementation science’s premises is analogous to the premise of contingency theories: context has critical implications for implementation. As such, contingency theories offer a well-established framework for conceptualizing the centrality of context, which implementation science deems of utmost importance.

Contingency theories suggest that best practices are contingent upon an organization’s internal and external contexts. Internal context is defined by factors that influence work activities from within an organization. Organizations that implemented SafeCare represented a diverse array of internal contexts, ranging from those found in government bureaucracies to foundations to small non-profits. Internal context affected stakeholders’ ability to make the changes necessary to implement SafeCare. For example, one CBO attempting to implement SafeCare was part of a large county health and human services agency. The agency’s multiple layers of bureaucracy limited the organization’s flexibility to take advantage of opportunities that might have facilitated SafeCare implementation. As described above, internal context also factored into the fit between SafeCare and CBOs’ existing processes; implementation was facilitated to the extent that SafeCare fit with an organization’s internal context [2] (e.g., was able to be integrated into a CBO’s electronic referral system; did not compete with existing programs). Contingency theories suggest that ICTs may have decreased uncertainty associated with resource constraints, thereby facilitating SafeCare implementation.

External context is defined by factors that exist outside and are not under the control of the organization, but which influence the organization’s structure and development. In some settings, ICTs allowed for tailoring SafeCare to the external context in which implementing organizations existed. For instance, among Latino populations in Southern California, ICTs implementing SafeCare were flexible enough to ensure fidelity to core SafeCare modules while translating SafeCare into Spanish and addressing other fundamental cultural differences (e.g., addition of information regarding home remedies that are common among Latino subcultures). In socially disadvantaged settings, ICTs also facilitated the tailoring of SafeCare to CBOs serving clients whose basic needs (e.g., food, shelter) superseded the needs that SafeCare addresses; in these settings, ICTs allowed the CBOs’ providers to prioritize meeting clients’ basic needs before initiating SafeCare [60].

#### Drawing on RDT to understand how CBOs’ external relationships facilitated SafeCare implementation

CBOs that implemented SafeCare depend on critical financial and human resources to support full implementation of the model. In particular, CBOs depended on the public child welfare agencies (and, in turn, counties and states) for the majority of financing to implement and deliver SafeCare. However, financing might have been low due, in part, to CBOs’ incentive to submit bids that are low enough to compete with the many other bids submitted. Also, not every CBO that bid on the contract was going to receive an award, heightening competition, and financial uncertainty for the CBOs that depended on these contracts. To buffer against the limited resources, and improve the chances that they would secure and maintain the contract, a small group of CBOs that were competing for the same bid collaborated with one another. Through this collaboration, the CBOs agreed that whichever organizations won the bid would pool the financial and other resources with the rest of the small group. Thus, the risk and rewards were shared among collaborative group members, and by doing so, additional CBOs implemented SafeCare beyond those that were contracted to do so. Thus, this collaborative agreement among CBOs helped to minimize resource constraints, and facilitated SafeCare implementation. However, although these CBOs implemented SafeCare, this collaborative arrangement came at the expense of the autonomy CBOs required to maintain relevance to local needs [2].

The emergence of this collaborative arrangement, which facilitated SafeCare implementation can be explained by RDT. Given the criticality of resources for organizational functioning, RDT suggests that the availability and access to resources (located in the external organizational environment) influences organizational behavior. Thus, organizational leaders make decisions to balance three competing needs: (1) dependence on others for resources, (2) maintenance of autonomy, and (3) the establishment of relationships [61]. Specifically, organizations develop partnerships, alliances, and other types of relationships to secure needed resources [62, 63]. However, these relationships create dependence—for instance, heavy fiscal dependence among private CBOs on the public child welfare agency—which can lead to a lack of certainty, predictability, and power among the local organizations especially amidst fluctuations in funding and procurement/allocation processes.

To counteract the consequences of dependence, organizations seek autonomy by attempting to control resources and environments [64]. Organizations exert control of their environment by placing their members on a board of directors, forming joint ventures or merging with or acquiring another organization, joining action sets of groups of organizations who make a collective response to external constraints, or engaging in other types of collaborative relationships. In the case of SafeCare, the interdependence associated with collaborative partnerships among CBOs (ICTs) may have been offset by the control that they gained over the financial resources available, their decreased dependence on the public child welfare agency, and reduced risks associated with the financial insecurity imposed by the new contracting procedures. Organizations that maintain autonomy are well positioned to influence their environments and maintain control over resources [61]. In fact, these administrative collaborations among competing CBOs, especially among those with a long history of trust and working together, are a strategy for adapting to or pushing back against competition [65, 66].

#### Organizational theories’ contribution to understanding SafeCare’s implementation

Viewed through the lens of contingency theories and RDT, the conclusions about SafeCare’s implementation may be enhanced and synthesized across studies. SafeCare researchers noted how their implementation study findings may have had limited generalizability to diverse contexts because some CBOs prioritized interventions that addressed client crises over using SafeCare [1] or used ICTs [57] to address local needs shaped by unique client demographics (i.e., American Indians in Oklahoma vs. Latinos in Southern California) [34], and competing child welfare programs [67]. Instead of conceptualizing these unique characteristics of diverse study contexts as limiting generalizability, contingency theories and RDT reframe these study “limitations” as strategic responses that align organizations with the external context. In the case of SafeCare, the use and effectiveness of ICTs for addressing barriers to implementation is explained by contingency theories, which suggest that active efforts are needed to address potential barriers to implementation due to idiosyncrasies associated with organizations’ internal and external contexts. The importance of interagency relationships for securing stable resources for implementation is explained by RDT, which suggests that interagency relationships, facilitated by trust, memoranda of understanding, and negotiations, were critical for CBOs attempting to balance dependence on funding agencies and SafeCare developers [57] with the autonomy they need to best implement SafeCare.

Linking SafeCare study results to organizational theory would allow authors to more explicitly contribute to generalizable knowledge regarding SafeCare implementation and, more broadly, to other EBIs. In essence, ICT was one approach to tailoring SafeCare to the local context and maintaining the autonomy needed to implement SafeCare in a way that was locally relevant in the face of dependence on funding agencies and SafeCare developers. In this way, findings regarding SafeCare’s implementation can be extrapolated to the implementation of any number of EBIs in any number of organizational contexts.

### Sustainment

Sustainment refers to the continuous use of an innovation, as intended, over time [40, 41]. In a study of 11 sites that implemented SafeCare, Willging et al. [50] found three levels of sustainment: (1) full sustainment (7 sites), with certified providers maintaining active SafeCare caseloads and regularly convened SafeCare teams (range of years sustained: 2–10); (2) partial sustainment (1 site), where certified providers used SafeCare in their active caseloads but did not regularly meet in SafeCare teams (years sustained: 4); and (3) not sustained (3 sites), where certified providers did not use SafeCare at the time of the study (range of years sustained: 1.5–2.3). Institutional theory and resource dependency theory help to explain variation in SafeCare sustainment across sites.

Willging et al. found that SafeCare sustainment was limited, in part, by policymakers who invested insufficient effort to develop the contracts necessary to sustain SafeCare [50]. Efforts to develop and maintain contracts have two functions. The first function can be explained with *institutional theory*: Efforts to maintain SafeCare contracts may have coerced CBOs to sustain SafeCare by establishing rules, regulations, and mandates set forth in contracts. The contracts also garnered support for SafeCare, creating normative pressure on CBOs to sustain SafeCare; in sites where SafeCare was not the assumed approach to preventing child abuse and neglect among child welfare social workers responsible for submitting referrals, SafeCare was not sustained. The second function can be explained with RDT: In essence, contracts confer the resources, including organizational relationships, needed for SafeCare sustainment. Indeed, policymakers could earmark funds for contracts that would support SafeCare and partner with collaborators to obtain sufficient resources for SafeCare sustainment [50].

Outside of contracts, Willging et al. also identified the train-the-trainer model associated with SafeCare as influential in its sustainment [50]. The benefits of the train-the-trainer model to SafeCare’s sustainment can be explained with RDT: The train-the-trainer model decreased CBOs’ dependence on SafeCare developers so that their staff could more autonomously sustain the practice, relying on and paying NSTRC less to sustain SafeCare.

#### Organizational theories’ contribution to understanding SafeCare’s sustainment

The conclusions that authors have drawn regarding SafeCare’s sustainment may be enhanced using the lens of the organizational theories described above. Authors have identified several specific determinants of SafeCare sustainment including the presence of champions, advocacy from policymakers, and relationships forged by policymakers. The mechanisms underlying these determinants can be explained with organizational theories, promoting their relevance for other EBIs; instead of encouraging future research to identify “strategies to promote congruence of leadership, mission and vision” [50], organizational theories suggest constructs that underlie the variables that authors have proposed as determinants of sustainment. Specifically, institutional theory suggests that organizations can harness coercive pressure (e.g., Oklahoma policymakers’ advocacy for SafeCare), mimetic pressure (e.g., champions for SafeCare), and normative pressure to sustain EBI use. RDT suggests that the relationships forged by policymakers that promoted SafeCare sustainment were a manifestation of organizations’ balance of dependence and autonomy to control the resources necessary for SafeCare use. In this sense, organizational theories are able to generalize determinants specific to a particular intervention (e.g., SafeCare) in a particular setting (e.g., Oklahoma), promoting the ability to predict determinants of sustainment in other contexts.

## Summary

This paper has demonstrated the application of four well-known organizational theories to retrospectively explain the implementation of SafeCare. More generally, this paper demonstrates the utility of organizational theory for explaining the influence of the complex interaction between organizations and their environment on implementation. Such explanations are largely lacking in extant implementation studies. There are a few notable exceptions. For example, studies have found institutional theory useful for explaining how an organization’s external environment influences the integration of evidence into practice [23] and which intervention components are most likely to be sustained [24]. TCE has been used to explain how organizational networks form [25] and why organizations engaged in pay-for-performance may “game” the system, inflating performance scores to maximize payment [26]. Shortell used institutional theory and TCE theory to understand the development and evaluation of accountable care organizations [27]. Invoking organizational theory more frequently to explain the influence of organizations’ interactions with their environment on implementation has the potential to advance knowledge in the field. For example, if transaction costs are found to influence organizations’ approaches to implementation, then implementation research and strategies can be designed to account for transaction costs.

### Prospective application of organizational theory to implementation research

Although most of this paper has addressed retrospective applications of organizational theory, organizational theories may also be used prospectively in implementation research. Table 2 displays applications of TCE, institutional theory, contingency theories, and RDT, as well as examples of hypothetical applications of these theories to SafeCare.

**Table 2:** Prospective application of organizational theory to SafeCare©

Organizational theories	Prospective application	Hypothetical prospective application to SafeCare
Transaction cost economics—explains how organizations decide whether to “make” a good or service internally or “buy” (i.e., acquire externally) a needed good or service.	Implementation may be outsourced if doing so is more efficient than implementing in-house. Implementation strategies should be selected to minimize transaction costs, which are positively associated with transaction frequency and uncertainty and asset specificity and likely negatively associated with implementation.	Bidding processes that minimize the frequency and uncertainty of the transactions required for SafeCare contracting will promote implementation by allowing organizations to commit resources to implementation instead of transaction costs.
Institutional theory—explains how organizations are motivated to align their structures and behaviors with the values, norms, and expectations espoused by institutions in their environments (e.g., government, client groups, accrediting bodies), as opposed to being primarily motivated by demonstrating superior outcomes or performance, to maintain legitimacy.	Implementation is likely to be influenced by coercive, normative, mimetic pressures from institutions within an organization’s environment. Implementation strategies should seek congruence with the values, norms, and expectations of relevant institutions in the implementing organization’s environment.	Staying attuned to the priorities of institutions in organizations’ environments will promote SafeCare implementation directly (e.g., institutions advocating for SafeCare implementation) and indirectly (e.g., organizations perceive institutions as approving of SafeCare implementation).
Contingency theories —explain how organizations’ actions are contingent upon an organization’s internal and external contexts, which influence the organizations’ structure and development.	Organizations’ structure is a critical determinant of implementation, and organizations’ structure continuously changes in response to dynamic internal and external contexts. Implementation strategies should be regularly revisited to address organizations’ dynamic internal and external contexts.	Designing SafeCare explicitly for adaptation will promote implementation by allowing organizations to accommodate their unique and dynamic internal and external contexts.
Resource dependency theory—explains how organizations structure themselves and associate with each other in order to acquire and maintain autonomy and the resources necessary to survive.	Implementation will be impeded to the extent that it limits organizations’ ability to acquire resources or maintain autonomy. Implementation strategies should address potential constraints of implementation on resource acquisition or autonomy.	Organizations will forge relationships to implement SafeCare insofar as doing so will yield resources that are worth more than the related decrease in autonomy.

Broadly, TCE helps to anticipate whether innovations will be implemented in-house or if implementation will be outsourced, which is especially applicable for studying implementation of EBIs within the public sector, since public agencies are encouraged to outsource services to private contractors. TCE suggests that implementation strategies should seek to minimize transaction costs. For example, TCE suggests that electronic handoffs of patient information among providers, such as the transmission of survivorship care plans among providers via electronic health records, will promote implementation [68]. TCE may also be helpful for informing how public agencies and other service funders design their procurement processes and contracts for EBIs.

Institutional theory suggests that an organization will implement an innovation when it is in congruence with the values, norms, and expectations espoused by institutions in an organization’s environment. Implementation strategies should either seek to influence the values, norms, and expectations of institutions within an organization’s environment or adapt the innovation to converge with institutional values, norms, and expectations to the extent possible with minimal compromise of fidelity to the intent of the innovation. This may involve, for example, framing an innovation using language that is likely to be perceived as aligned with institutions’ values, norms, and expectations.

Contingency theories suggest that implementation optimally involves adaptation to organizations’ internal contexts (e.g., organizational capacity, mission and values, culture) and external contexts (e.g., patient needs, political environment, geographic locations) and to changes in those contexts over time. This suggests that innovations should be developed with a dynamic approach to implementation in mind. A common elements approach to implementation would potentially be most successful: implementing common techniques across EBI models would allow clinicians greater flexibility to meet diverse client needs and organizations more flexibility in training their workers [69, 70]. To optimize intervention delivery, scholars have advocated for efforts to systematically adapt interventions to the varied and unique contexts in which they are disseminated [71]. Evidence regarding successful adaptation of promising interventions to a variety of settings continues to accumulate. For example, a diabetes care quality improvement initiative was originally successfully launched in an insured (health maintenance organization) setting and later adapted to meet the needs of federally qualified health centers [72].

RDT suggests that organizations will weigh the resources to be gained against the autonomy to be lost from innovation implementation. To the extent that organizations can use strategies to tip the balance in their favor, implementation may be more successful. This is the contention of the “systems” thinking/change perspective [73], which asserts that successful implementation is a function of the control key stakeholders have over resources. Empirical evidence supports this hypothesis. For example, autonomy was found to be a key determinant of the implementation of an intervention to promote effective pain management in older people [74].

Our paper has several limitations. First, the organizational theories that we have described in this paper themselves have limitations. Perrow critiqued TCE’s neglect of power relations. Indeed, in some cases, organizational theories’ propositions sometimes contradict each other [75]. For example, TCE may predict that an organization will fail to adopt an EBI due to excessive transaction costs, whereas institutional theory may predict that the organization will adopt the EBI to gain legitimacy, despite high transaction costs. Underlying these differences is divergence in the theories’ paradigms. TCE, contingency theories, and RDT’s paradigms are rooted in economics, which values rationality to explain organizational outcomes. In contrast, institutional theory takes a more sociological view and emphasizes the role of social forces, relationships, and norms in explaining organizational outcomes. Also, in contrast to frameworks, which are often intended to be comprehensive of determinants of implementation, theories tend to focus on a limited group of constructs about which specific causal relationships are proposed [76]. Further, in this paper, we have applied organizational theories to understanding macro-organizational relationships (i.e., relationships between organizations and their external environments). These theories may also be useful for understanding micro-organizational relationships, i.e., how factors within organizations (e.g., culture, climate, leadership) influence implementation. Theories in the field of organizational behavior may also be useful for understanding micro-organizational relationships. As an example, researchers have extended theories about organizational citizenship behavior to implementation science by developing an implementation citizenship behavior scale, which assesses employee behaviors that go beyond what is required to implement EBIs. Additionally, Aarons and colleagues have used leadership and strategic climate theories to derive an implementation leadership scale, allowing researchers to examine the impact of strategic leadership on organizational context for implementation. Health psychologists have increasingly applied behavior change theories to explain the behavior of individuals including providers and patients. For example, Karvinen et al. used the theory of planned behavior to understand exercise among endometrial cancer survivors [77]. Research that attempts to demonstrate causal relationships between determinants at multiple levels of the ecological framework and implementation may benefit from applying theories in several of these fields.

Second, in this paper, we have presented four organizational theories that may be useful in implementation research because they were particularly apt for explaining SafeCare’s adoption, implementation, and sustainment. Other organizational theories that may be useful in implementation research include population ecology of organizations, agency theory, and exchange theory. For example, population ecology suggests that an organization’s adaptive need for reliability and accountability to survive predisposes it to inertia and resistance to the change required to implement innovations [78]. Principal-agent and stewardship theories might be useful for investigating the influence of approaches for managing contractual relationship for implementation given the trend toward privatization and contracting in health and human services [79].

Third, in this paper, we apply organizational theory in a qualitative fashion; this diverges from the largely quantitative application of organizational theory in other areas of health services research (e.g., [80]). Our retrospective, qualitative application of organizational theory provides an explanation for SafeCare implementation; however, we do not test hypotheses derived from TCE, RDT, institutional theory, or contingency theories. In contrast, quantitative approaches or deliberately designed qualitative studies might be more conducive to testing hypotheses derived from the application of these four theories to implementation.

Despite these limitations, our paper has important implications for implementation strategies, the evaluation of implementation efforts, measurement, research design, theory, and practice.

Implementation strategies consist of practical approaches to promote EBI adoption, implementation, and sustainability [81]. Domains of implementation strategies include planning, education, financing, restructuring, quality management, and attending to the policy context [82]. Applying organizational theory to implementation strategies helps to explain why these strategies promote implementation and may promote more effective strategy selection. For example, TCE suggests that quality management may promote implementation by decreasing transaction costs associated with desired behaviors. TCE also encourages implementation practitioners to account for the transaction costs associated with a given strategy; the transaction costs associated with resource-intensive restructuring, for example, may outweigh its potential benefits to implementation. Institutional theory suggests that some strategies may have objective benefits to implementation as well as conferring legitimacy on the organization implementing the EBI. For example, the legitimacy associated with engaging in quality management, regardless of its objective benefits, may be sufficient to use such a strategy. Contingency theories suggest that strategies are not one-size-fits-all—organizations must select and tailor strategies to their internal and external contexts—a perspective that has been advocated to promote implementation strategies’ effectiveness [83]. RDT suggests that some strategies may be successful in promoting implementation by maintaining a balance between autonomy and dependence on others. For example, education of staff may act as a mechanism for gaining autonomy from EBI experts. Organizational leaders may benefitting from select implementation strategies with a desired balance between autonomy and dependence on other organizations in mind.

Organizational theory also has implications for implementation evaluation. Increasingly, theory-driven evaluation (e.g., [84]), such as process analyses and N-of-1 trials, complement traditional randomized controlled trials of implementation outcomes by suggesting mechanisms underlying the relationship between an EBI and its outcomes [85]. Organizational theory suggests that transaction costs; needs for autonomy, resources, and legitimacy; and internal or external contexts may drive EBI outcomes. Drawing on organizational theories in implementation research will require development of measures such as institutional pressures (coercive, normative, and mimetic) [46, 47]; resource complexity, availability, and stability that influence organizational dependence/autonomy (e.g., [86]); and the uncertainty, asset specificity, and transaction costs (e.g., [87]) associated with organizational “make vs. buy” decisions. These and other constructs characterizing the external organizational environment have been somewhat absent in prior implementation studies [88] and limited in the larger organizational literature as well [87] due to definitional ambiguity and imprecision [89]. Clear definitions to guide measurement development will be critical, as well as examination of the merits of using objective versus perceived measures of the context [90]. Although objective measures are generally preferred, organizational leaders make decisions based on how they perceive the context, and therefore, perceptual or self-report measures might also be valid [91]. Finally, scholars will need to carefully consider the unit of analysis, for instance, whether the institutional or resource environments can be captured via aggregated organizational reports.

Implementation researchers who use the lens of organizational theory will likely need to adjust their approach to design. Applying organizational theory often (but not exclusively, in the case of RDT, for example) implies analysis at the organizational level. Particularly for quantitative studies, such as experiments or quasi-experiments, recruiting a number of organizations to achieve sufficient power may be challenging. In contrast to studies at the provider or individual level, in which participants may be difficult to recruit but are plentiful, organizations both are often difficult to recruit and are scarce, relative to individuals. For observational, qualitative studies, relatively small numbers of organizations may be sufficient to achieve study objectives. However, organizational policies and the process of consenting an organization can make study recruitment challenging. For example, recruiting organizations requires an agent of the organization—typically a top leader—to consent on behalf of the organization; in many cases, organizational research requires the participation of employees whose responses are aggregated to the organizational level, and organizational consent does not imply employees’ consent [92]. Research at the organizational level may also require longer study periods since organizational change, including success or failure outcomes, is often slow [78]. We recommend that implementation researchers who wish to incorporate organizational theory into their work collaborate with researchers who have expertise in organizational theory and research (often found in the fields of sociology, public administration, political science, and management). Implementation researchers who would like to apply organizational theory may find useful introductory texts on organizational theory [16, 93] and conferences that feature studies that incorporate organizational theory, such as the Association for Research on Nonprofit Organizations and Voluntary Action [94], the Health Care Division of the Academy of Management [95], and the Organizational Theory in Health Care Association [96]. We also recommend that implementation research training programs, including doctoral programs and postdoctoral training programs such as the Training Institute on Dissemination and Implementation Research in Health and the Implementation Research Institute, incorporate formal training in organizational theory. Other fields’ (e.g., public administration [97–99]) success incorporating organizational theory suggests that doing so is feasible. Indeed, establishing common ground in organizational theory may serve to promote collaborations with these fields, which are currently lacking [100].

Our paper also suggests implications for theory. Broadly speaking, organizational theories propose specific manifestations of organizations’ efforts to survive [78]: To survive, organizations minimize costs; adhere to values, norms, and expectations of institutions within their environment; develop characteristics that differentiate them to compete with others; and acquire resources and autonomy. Organizations implement innovations to survive—to comply with accrediting bodies [101], to respond to changing patient needs [102], to offer appealing services in-house [103]. In this sense, implementation may be a determinant of organizational survival; to the extent that implementing an innovation does not promote survival, implementation is unlikely to occur. Additionally, examining the barriers to implementation offers an opportunity to investigate overlap across theories and to advance theories that specify and explain cross-level influences (e.g., influences that span across organizational environment, organizational behavior, and provider behavior).

Our paper also suggests that key organizational theories should be incorporated into implementation frameworks. Developers of the Consolidated Framework for Implementation Research (CFIR) did not draw upon the kinds of classic organizational theories that we advocate for implementation research, but the CFIR includes constructs that relate to some key organizational theory constructs [12]. For example, the CFIR’s cosmopolitanism domain (i.e., the extent to which an organization is networked with others) suggests the importance of interagency relationships. RDT provides a rationale for including this construct: Organizations with stronger networks may facilitate implementation by increasing access to resources including information, influence, and funding [62]. Drawing on organizational theory, we can further specify and expand upon constructs included in implementation frameworks.

Finally, the application of organizational theories to implementation has practical implications. Organizational theories suggest that managers should be aware of influences from the external environment that may influence adoption, implementation, and sustainment. TCE, for example, suggests that having established relationships with external organizations that influence implementation may promote implementation success. Institutional theory suggests that gaining legitimacy—regardless of whether it improves performance—may drive adoption decisions. In some cases, the drive to gain legitimacy may undermine the objectives of an implementation effort; organizational leaders may superficially implement an EBI, without effecting change, to conform to institutional pressures. Contingency theories suggest that successful implementation will likely require adaptation of an intervention to the manager’s unique organizational context. RDT suggests that threats to maintaining autonomy or resources represent threats to implementation and sustainment. Organizational theory also encourages a skepticism of practice trends. In response to practice trends, researchers often assess strategies for accommodating these trends. In contrast, organization theory can help to alert practitioners and researchers to potential negative consequences of practice trends. For example, institutional theory suggests that following practice trends may confer legitimacy but not necessarily performance improvement.

## Conclusions

Organizational theories offer implementation researchers a host of existing, highly relevant, and heretofore largely untapped explanations of the complex interaction between organizations and their environment. Researchers and practitioners may benefit from the insights into implementation that organization theories offer.

## Abbreviations

BV-PIPS: Best Value-Performance Information Procurement System
CBO: Community-based organization
CFIR: Consolidated Framework for Implementation Research
EBI: Evidence-based intervention
ICT: Interagency collaborative team
NSTRC: National SafeCare Training and Research Center
RDT: Resource dependency theory
TCE: Transaction cost economics
